# Supplementary material for: Photochemistry of the pyruvate anion produces CO2, CO, CH3–, CH3, and a low energy electron
Source: Nat Commun. 2022 Feb 17;13:937. doi: 10.1038/s41467-022-28582-4 (PMC8854594; doi:10.1038/s41467-022-28582-4)
Supplement: Supplementary file 1 — Supplementary information [file 41467_2022_28582_MOESM1_ESM.pdf]

Supplementary Information:

Photochemistry of the pyruvate anion produces CO_2, CO, CH_3^-, CH_3, and a low energy electron

Connor J. Clarke, Jemma A. Gibbard, Lewis Hutton, Jan R. R. Verlet*, Basile F. E. Curchod*

Department of Chemistry, Durham University, Durham DH1 3LE, United Kingdom

j.r.r.verlet@durham.ac.uk; basile.f.curchod@durham.ac.uk

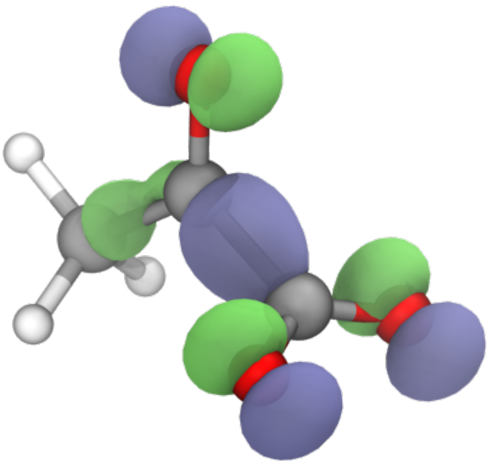

Supplementary Figure 1: Dyson orbital for the photodetachment of the pyruvate anion from its S_0 (anionic) ground state to its D_0 (neutral) ground state, obtained at the (U)DFT/ωB97X-D/aug-cc-pVDZ level of theory.

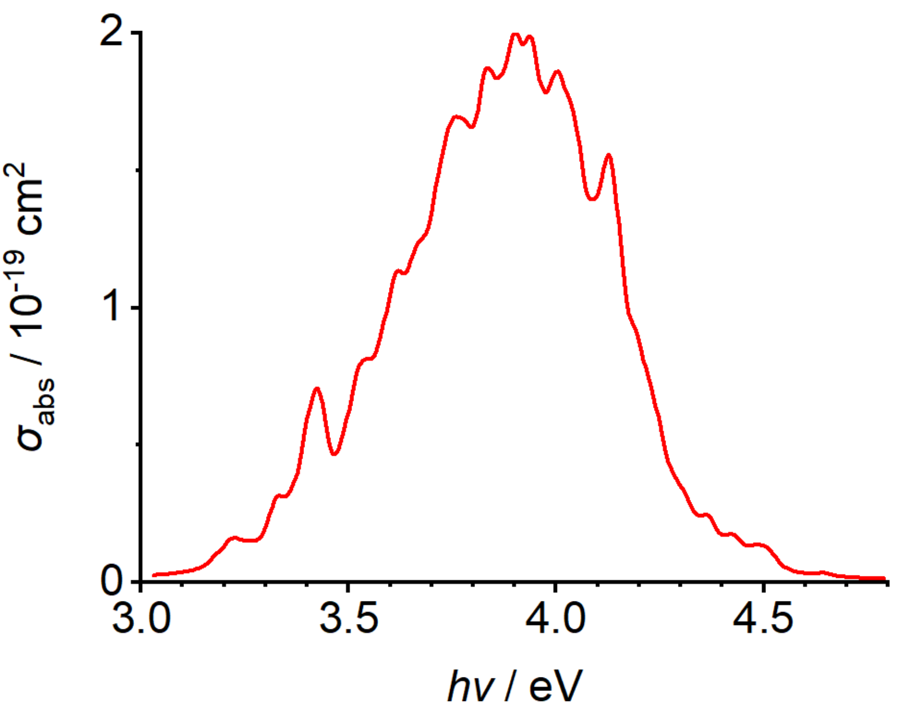

Supplementary Figure 2: Calculated photoabsorption cross-section for the pyruvate anion. The NEA has been employed, constructing a Wigner distribution from the S_0 minimum-energy geometry and corresponding frequencies obtained with DFT/ωB97X-D/aug-cc-pVDZ. The transition energies and oscillator strengths for each of the 500 sampled geometries were calculated with SCS-ADC(2)/cc-pVTZ. Only the transition to the first singlet state ($n\pi^*$) was considered for each sampled geometry. Additional information can be found in the Computational Details.

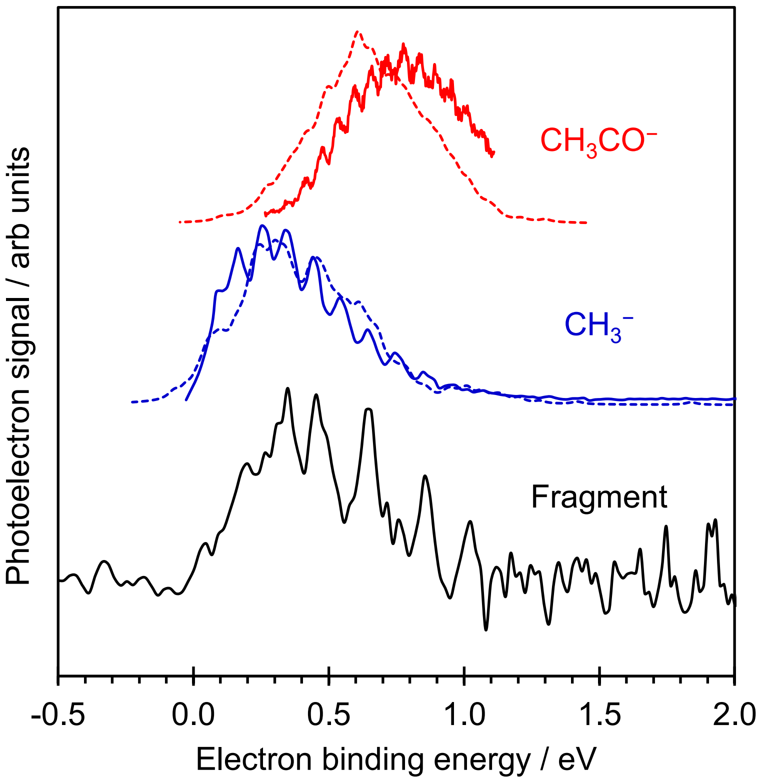

Supplementary Figure 3: Comparison of photoelectron spectrum of fragment with acetyl and methide anions. Reproduction of Figure 3(b) from manuscript (bottom, black line) compared to the measured photoelectron spectra of CH_3^- (middle, blue line) and CH_3CO^- (top, red line). All spectra are offset for clarity. The CH_3^- is digitised and reproduced with permission from: Ellison, G. B.; Engelking, P. C.; Lineberger, W. C. *J. Am. Chem. Soc.* **1978**, 100, 2556. Copyright 1978 American Chemical Society. The CH_3CO^- spectrum is digitised and reproduced with permission from: Nimlos, M. R.; Soderquist, J. A.; Ellison, G. B. *J. Am. Chem. Soc.* **1989**, 111, 7675–7681. Copyright 1989 American Chemical Society. The calculated photoelectron spectra for CH_3^- and CH_3CO^- are indicated by dashed lines.

Supplementary Note 1: Photon flux dependence of methide emission

The dependence of the methide peak on photon flux, Φ, was measured, with data presented below (red solid circles) as a log-log plot of signal (integrated PE signal of the methide peak normalised to the one-photon direct detachment peak) versus laser power (in mW). The gradient (black line) is expected to equal the number of photons absorbed for a multi-photon transition. From the Figure below, we find a gradient of 1.047, suggesting that the methide peak is formed by a one-photon process. While this is inconsistent with the stated two-photon process, we can readily rationalise this inconsistency. The power-dependence is only valid when the cross-sections associated with the two separate excitations processes is the same. This is not the case here as the first photon excites pyruvate ($S_1 \leftarrow S_0$), while the second photon detaches an electron from methide. There is no reason to expect the cross-sections for these to be similar. If either cross-section is significantly larger than the other, then that transition will dominate, and the photon flux dependence will appear to have a gradient of 1 in the log-log plot as observed.

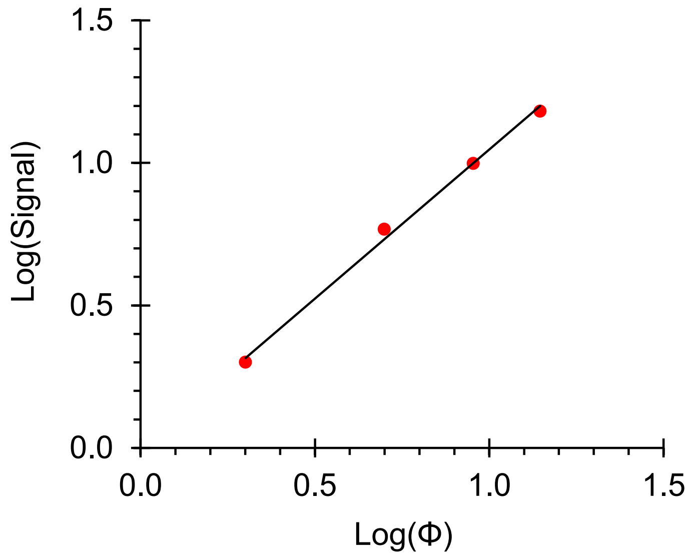

Supplementary Figure 4: Dependence of the relative integrated photoelectron signal of the methide peak on photon flux.
